# Investment on Education of Future Parents: the Best Measure for Enhancement of a Community Disaster Resiliency

**DOI:** 10.5812/ircmj.10349

**Published:** 2014-05-05

**Authors:** Behnaz Rastegar Far, Mohammad Javad Moradian, Ali Ardalan, Javad Babaie

**Affiliations:** 1 Disaster and Emergency Management Center, Shiraz University of Medical Sciences, Shiraz, IR Iran; 2 Department of Disaster Public Health, School of Public Health, Tehran University of Medical Sciences, Tehran, IR Iran; 3 Department of Disaster and Emeregncy Health, National Institute of Health Research, Tehran University of Medical Sciences, Tehran, IR Iran; 4 Harvrad Humanitarian Initiative, Harvard School of Public Health, Boston, USA

**Keywords:** Residence Characteristics, Disasters, Resilience, Psychological

A disastrous situation means a condition which becomes difficult to cope with for human being and there is less accommodation especially during the early phases of response. During last decades, the paradigm of disaster management has been shifted from response to prevention and preparedness for disasters. In addition, the governments have realized that they hardly are capable to stand alone against the disasters and they need communities alongside for better disaster risk reduction and response. This was evident in recent disasters even in developed countries, like the super storm Sandy (2012) and the Japan tsunami and earthquake (2010). We witnessed the same situation in Iran’s disasters, e.g., Bam (2003), Zarand (2005), Lorestan (2006), East Azerbaijan (2012) earthquakes (1) and Golestan flash floods (2001 and 2005). These events revealed the crucial role of communities for better response and faster recovery. We also have learned that we need to invest on the community resiliency before a disaster happens, i.e. enhance the capability of the community to bounce back as soon as possible based on its own recourses (2).

What are the best measures for enhancing the community resiliency? To answer this critical question, we should keep this fact in mind that building a resilient community requires a long term investment on the education and safety culture. Primary and secondary schools are the best places to be targeted for this purpose. While the parents are busy with daily life, the school children are able to convey the educational messages and methods of participatory vulnerability and capacity assessments from schools to their own family (3). They are also the next generation of parents and responsible for education of their own children.

In line with the Hyogo Framework for Action and the UN Decade for Education and Sustainable Development (2005-2014), as well as continuing work by governments and other actors toward achievement of the Millennium Development Goals, we wish to reemphasize on the strategy of children education for disaster preparedness and resiliency. Our future resiliency for disasters depends on investment on education of today’s children. This can be done using a properly designed simple intervention program for schools. 
